# Regulation of focal adhesion turnover by ErbB signalling in invasive breast cancer cells

**DOI:** 10.1038/sj.bjc.6604901

**Published:** 2009-02-03

**Authors:** Y Xu, N Benlimame, J Su, Q He, M A Alaoui-Jamali

**Affiliations:** 1 Department of Medicine, Lady Davis Institute of the Sir Mortimer B. Davis Jewish General Hospital, Segal Comprehensive Cancer Center, McGill University, Montréal, Canada; 2 Department of Oncology, Lady Davis Institute of the Sir Mortimer B. Davis Jewish General Hospital, Segal Comprehensive Cancer Center, McGill University, Montréal, Canada

**Keywords:** breast cancer, ErbB2/Her-2, focal adhesion, cell invasion, metastases

## Abstract

A crucial early event by which cancer cells switch from localised to invasive phenotype is initiated by the acquisition of autonomous motile properties; a process driven by dynamic assembly and disassembly of multiple focal adhesion (FA) proteins, which mediate cell–matrix attachments, extracellular matrix degradation, and serve as traction sites for cell motility. We have reported previously that cancer cell invasion induced by overexpression of members of the ErbB tyrosine kinase receptors, including ErbB2, is dependent on FA signalling through FA kinase (FAK). Here, we show that ErbB2 receptor signalling regulates FA turnover, and cell migration and invasion through the Src–FAK pathway. Inhibition of the Src–FAK signalling in ErbB2-positive cells by Herceptin or RNA interference selectively regulates FA turnover, leading to enhanced number and size of peripherally localised adhesions and inhibition of cell invasion. Inhibition of ErbB2 signalling failed to regulate FA and cell migration and invasion in cells lacking FAK or Src but gains this activity after restoration of these proteins. Taken together, our results show a regulation of FA turnover by ErbB2 signalling.

The ability of invasive tumour cells to invade surrounding tissue structures at primary sites requires tumour cell capacity to become motile and invasive. This process involves a series of cellular events, which includes formation and extension of protrusions in response to chemotactic signals, formation of stable cell focal adhesion (FA)–matrix attachment near the leading edge of the protrusions, and movement of the cell body forward aided by the release and retraction of the FAs at the trailing edge of the cell. Therefore, FAs are established as key regulators of cell motility and cell invasion since they can act as signalling centres and provide robust anchors to the extracellular matrix (ECM), which represent traction points for the generation of cell tension and motility.

Several protein kinases and phosphatases appear to be central to the regulation of adhesion turnover and stability, including the FA kinase (FAK). We have reported that ErbB2-induced cell invasion is dependent on FAK signalling (Benlimame *et al*, 2005). Focal adhesion kinase is a key multifunctional adaptor and signalling molecule that has been shown to play a role in FA formation and turnover and is required for cell motility and cell invasion (Ilic *et al*, 1995; Sieg *et al*, 2000). In motile cells, FAK along with other partners of the FA network is recruited to membrane cell protrusion following integrin engagement and/or receptor activation, where it becomes autophosphorylated at tyrosine Y397. Src is then recruited through its SH2 domain to the phosphorylated FAK, and both enzymes subsequently phosphorylate additional FAK tyrosine residues in the catalytic and C-terminal domains creating additional docking sites for SH2-containing proteins (Schaller *et al*, 1994; Calalb *et al*, 1995; Parsons *et al*, 2000). Similar to FAK, Src also regulates FA dynamics and both FAK- and Src-deficient fibroblasts have motility defects and stable FAs attributed to defective FA turnover required for cell locomotion.

In this study, we established a functional role for ErbB signalling in the regulation of FA turnover in invasive cells through the Src–FAK pathway, and provided a novel mechanism for the anti-ErB2 antibody Herceptin in inhibiting FA turnover and preventing the early stage cancer invasion.

## Materials and methods

### Antibodies and reagents

Antibodies for ErbB2 and Src were from Oncogene (Boston, MA, USA). Antibodies to Akt1, phospho-Akt (Ser473), ERK1/2, and phospho-ERK (Tyr202/Tyr204) were from Cell Signaling Technology (Beverly, MA, USA). Antibodies to phosphotyrosine and ERK2 were from Upstate Biotechnology (Waltham, MA, USA). Antibodies to RhoA and phospho-FAK-Y925 were from Santa Cruz Biotechnology (Santa Cruz, CA, USA). Antibodies to FAK, phospho-FAK-Y397, and phospho-FAK-Y861 were from Biosource International (Camarillo, CA, USA). Antibody to vinculin was from Sigma-Aldrich (St Louis, MO, USA). Antibody to GAPDH was from Cedarlane (Burlington, Ontario, Canada), and antibody to *α*-tubulin was from Abcam (Cambridge, MA, USA). Heregulin *β*-1 (HRG *β*1) was from Neomarkers (Fremont, CA, USA). Fibronectin and nocodazole were from Sigma-Aldrich. Taxol and Herceptin were from the Oncology pharmacy of the Jewish General Hospital.

### Cell culture

Cells MCF-7, MDA-231, T47D, and BT20 were obtained from ATCC and maintained in RPMI 1640 (Mediatech, Washington, DC, USA). SKBR3 cells were maintained in McCOY's medium (Wisent Inc. St-Bruno, Quebec, Canada). Mouse embryonic fibroblasts FAK proficient (FAK^+/+^) and FAK deficient (FAK^−/−^) were provided by Dr Dusko Ilic (University of California, San Francisco, CA, USA) and maintained as described by Ilic *et al* (1995). SYF cells (Src, Yes, and Fyn triple knockout) and Src reconstituted SYF cells were from the ATCC, and were maintained in DMEM. All the media were supplemented with 10% FBS and penicillin and streptomycin antibiotics.

ErbB2 receptor was overexpressed using a bicistronic retrovector that coexpresses ErbB2 receptor with the enhanced green fluorescent protein (EGFP) as described earlier (Yen *et al*, 2002). Focal adhesion kinase reconstitution in FAK-deficient cells was achieved by the expression of pBabe-GFP-mFAK-puro retroviral particles (kindly provided by Dr D Schlaepfer, the Scripps Institute) as described earlier (Bergeron *et al*, 2000).

### Generation of cells expressing siRNA FAK and siRNA Akt-1

Stable FAK and Akt1 knockdown cells were generated in a polyclonal population as described by Benlimame *et al* (2005). Focal adhesion kinase-1: gcatgtggcctgctatgga; FAK-2: gccttaacaatgcgtcagt; Akt1-1: ggagatcatgcagcatcgc; Akt1-2: acgaggggagtacatcaagtc are the target sequences used for stable knockdown of FAK and Akt1.

### Cell proliferation assay

Cells (1 × 10^3^) were seeded in 96-well plates and incubated for 16 h. In the presence of mouse IgG1 as control or 5 *μ*g ml^−1^ Herceptin, cells were then treated with 10 ng ml^−1^ heregulin. Cell survival was evaluated 96 h later using the 3-(4,5-dimethylthiazo-2-yl)-2, 5-diphenyltetrazolium bromide (MTT) metabolic assay as described earlier (Yen *et al*, 2002).

### Immunoprecipitations and western blot analysis

Immunoprecipitation and western blot were carried out as we described earlier (Benlimame *et al*, 2005). Blots were detected using appropriate antibodies and visualised by enhanced chemiluminescence detection.

### RhoA GTPase activation assay

Cell extracts were collected from nocodazole-treated MDA231-ErbB2 cells immediately after 4 h nocodazole treatment (0 min) and 60 min after growth in nocodazole-free medium, in the presence of mouse IgG1 as control or 5 *μ*g ml^−1^ Herceptin. Activated RhoA-GTPase level was determined by immunoprecipitation using the rhotekin-binding domain (Ren *et al*, 2000) coupled to western blot analysis using anti-RhoA. Whole cell lysates were reprobed for total RhoA.

### FAK and Src kinase assays

Cells at 80% confluence were serum-starved for 24 h, pretreated with either 5 *μ*g ml^−1^ Herceptin or IgG1 in serum-free medium and then stimulated with 10 ng ml^−1^ heregulin for the indicated time. Cells were washed with cold PBS, and 500 *μ*g of total cell lysate were used for immunoprecipitation with FAK antibody. Focal adhesion kinase assay was performed as described by Sieg *et al* (2000). Src kinase assay was performed based on a protocol described by Nagata *et al* (2004).

### Focal adhesion disassembly assay

Assay for FA disassembly and reformation after nocodazole treatment has been described earlier (Ezratty *et al*, 2005). Serum-starved cells were grown on glass coverslips and incubated with Herceptin (5 *μ*g ml^−1^) or mouse IgG1 for 1 h followed by 10 *μ*M nocodazole treatment for 4 h. Nocodazole was washed out with serum-free medium, cells were fixed and stained with vinculin, phospho-FAK-Y397, and/or *α*-tubulin.

### Indirect immunofluorescence analysis

Cells were seeded on fibronectin-coated coverslips for 2 days at a density of 20 000–50 000 cells per 35 mm dish, incubated for 24 h in serum-free medium and then pretreated with 5 *μ*g ml^−1^ Herceptin or mouse IgG1 before adding 10 ng ml^−1^ HRG *β*1. The cells were fixed, permeabilised, labelled for immunofluorescence and analysed using florescence microscopy as we described earlier (Yen *et al*, 2002; Benlimame *et al*, 2005).

### Invasion and cell migration assay

Cell invasion experiments were performed with 8-*μ*m porous chambers coated with Matrigel (Becton Dickinson, Franklin Lakes, NJ, USA) according to the manufacturer's recommendations. Cell migration was assayed using the qualitative wound-healing assay as described earlier (Benlimame *et al*, 2005).

### Time-lapse video microscopy

For live fluorescent imaging, the BT20 cells were stably transfected with EYFP-paxillin and plated in RPMI-1640 on a multi-well Chambered coverglass (Lab Tek, nunc™, Rochester, NY, USA) coated with 10 *μ*g ml^−1^ fibronectin for 1 h. Before filming, media was replaced with complete RPMI-1640 media supplemented with either IgG1 control or 5 *μ*g ml^−1^ Herceptin in different wells. Cells were placed directly on a heated stage and supplemented with 5% CO_2_. Fluorescent images were captured every 1 min for 2 h using a heated × 100/1.40NA objective at the optimised Nipkow Spinning Disk confocal microscope (Quorum Technologies Inc., Guelph, Ontaria, Canada). Cooled CCD camera control and image acquisition was done using Volocity imaging software (Improvision, Waltham, MA, USA). At the extremely high speed, we investigated different cells in less than 10 s interval. The experimental results show representative cells from a minimum of five different experiments. Fluorescence intensities of individual adhesions from background-subtracted images were measured over time using volocity imaging software. Measurements were made on at least 25 individual adhesions in five separate cells for both control IgG1- and Herceptin-treated cells. Data are presented as mean±s.d. Statistical significance was analysed using Student's *t*-test.

## Results

### ErbB signalling modulates cell invasion in an FAK-dependent manner

**Figure 1:**
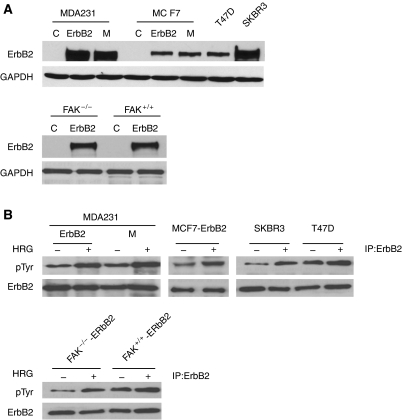
Overexpressed ErbB2 receptor is activated by heregulin *β*1 in the cancer cell models used. (**A**) Total cell lysates were isolated from parental cells (C), ErbB2 overexpressing cells (ErbB-2), and a metastatic cell variant selected *in vivo* from a lung metastatic nodule induced by MDA231-ErbB2 cells implanted into the mammary fat pad (MDA231-M). Proteins were resolved by SDS–PAGE and immunoblotted with anti-ErbB2 antibody. As an internal control GAPDH was used. (**B**) Cells were serum-starved for 24 h and then stimulated with 10 ng ml^−1^ HRG *β*1 for 10 min. ErbB2 was immunoprecipitated from total cell lysates, resolved by SDS–PAGE, and blots were probed using a phosphotyrosine antibody and reprobed with antibody against ErbB2, as described in Materials and Methods.

To address the impact of ErbB signalling on cell invasion, we used a panel of human breast cancer cell models overexpressing exogenous ErbB2 (MCF7 and MDA231), endogenous ErbB2 (SKBR3 and T47D), or MDA231 cell variant (MDA231-M) selected *in vivo* from a metastatic lung nodule induced by MDA231-ErbB2 cells after implantation into the mammary fat pad. In addition, mouse embryonic fibroblasts proficient (FAK^+/+^) or deficient (FAK^−/−^) in FAK and overexpressing ErbB2 were used. ErbB2 was overexpressed using a retroviral system that expresses both the EGFP and the receptor (Benlimame *et al*, 2005). Figure 1 summarises the expression status of ErbB2 in these cells (Figure 1A) and following receptor activation with 10 ng ml^−1^ HRG *β*1. Heregulin *β*1 is the ligand for ErbB-3 and ErbB-4, which activates ErbB2 by inducing receptor heterodimerisation and transphosphorylation. As noted, the basal phosphorylation level of ErbB2 is increased even in the absence of ligand stimulation (Yarden and Sliwkowski, 2001). As shown in Figure 1B, in all cell lines tested, ErbB2 is autophosphorylated in cells overexpressing the receptor, and exposure to HRG *β*1 further enhances receptor phosphorylation. In the parental cells used to overexpress ErbB2, the levels of ErbB2 protein and receptor phosphorylation ranged from being very weak to undetectable (Yen *et al*, 2002; Benlimame *et al*, 2005).

**Figure 2:**
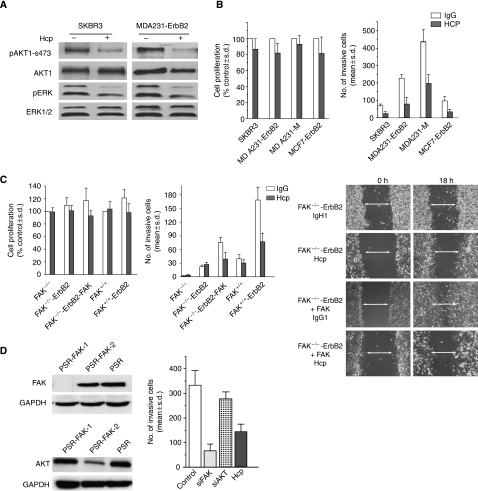
Herceptin induces downregulation of Akt and ERK signalling in ErbB2-positive cells, and inhibits cell invasion in a FAK-dependent manner. (**A**) Impact of Herceptin (Hcp) (+, 5 *μ*g ml, 48 h) or control IgG1 (−, 5 *μ*g ml^−1^, 48 h) on the expression and phosphorylation levels of Akt1 and MAPK-ERK in MDA231-ErbB2 and SKBR3 cells using western blot on total cell extracts. (**B**) Left panel: the rate of cell proliferation was similar between Herceptin-treated and control IgG1-treated cells using the MTT assay. Cell proliferation (expressed as % of control cells) was monitored by the MTT assay. Results are expressed as the average of four independent experiments±s.d. Right panel: herceptin inhibits cell invasion on the Boyden chamber invasion assay. Results are expressed as the mean of at least three experiments and three fields per condition±s.d. (**C**) FAK-deficient (FAK^−/−^-ErbB2) and FAK-proficient (FAK^+/+^-ErbB2) cells were used to show the essential role of FAK in Herceptin-induced inhibition of cell proliferation and cell invasion using similar conditions described in (**B**). Results are expressed as mean±s.d. Figure on the right is a representative wound-healing experiment on ErbB2-FAK-proficient, ErbB2-FAK-deficient, and ErbB2-FAK-reconstituted cells treated with IgG1 or 5 *μ*g ml^−1^ Herceptin. Note the requirement of FAK for Herceptin-induced inhibition of cell migration. (**D**) Left panel: representative western blots showing FAK and Akt1 expression in MDA231-ErbB2 cells stably engineered to express FAK siRNA or Akt1 siRNA using pSuper-retro puro vector. Control cells expressed retroviral vector pSuper-retro-puro alone (PSR). As an internal control GAPDH was used. Right panel: cell invasion was examined on MDA231-ErbB2 cells expressing PSR (control), FAK siRNA, Akt siRNA, or treated with 5 *μ*g ml^−1^ Herceptin as described in (**B**) (legend for the right panel). For each condition, the experiments was performed three times and the results are expressed as mean±s.d.

To address the impact of ErbB2 signalling on FAs, cell invasion, and cell proliferation, we examined the impact of ErbB2 signalling modulation by Herceptin. We initially confirmed the responsiveness of ErbB2-positive cells to Herceptin focusing on the MDA231-ErbB2 and SKBR3 cell lines. In this case, cells were exposed to 5 *μ*g ml^−1^ Herceptin and monitored for the activation of Akt1 and mitogen-activated protein kinase (MAPK), Rapid Akt1 and MAPK dephosphorylation following Herceptin treatment as reported earlier (Ignatoski *et al*, 2000; Yakes *et al*, 2002; Nagata *et al*, 2004). As shown in Figure 2A, Herceptin inhibited active MAPK and active Akt1 as measured by antibodies specific to phospho-MAPK and phospho-Ser473 Akt1, respectively.

Cells were then serum-starved and stimulated with HRG *β*1, in the presence of mouse IgG1 or 5 *μ*g ml^−1^ Herceptin. Cell proliferation was determined by the MTT assay. Cell invasion experiments were performed using the Matrigel Boyden chamber assay. HRG *β*1 was added into the lower compartment of the chamber as a chemotactic reagent. As shown in Figure 2B, ErbB2 modulation by Herceptin had a modest inhibitory effect on the proliferation of ErbB2-positive cell lines, and no significant cell death was observed after 72–96 h cell exposure to Herceptin (not shown). In contrast, Herceptin treatment induced a potent reduction in the invasion of ErbB2+ cells (*P*<0.005, compared with control cells).

To address the importance of FAK on cell invasion following ErbB2 modulation, we compared Herceptin activity in FAK-proficient and FAK-deficient mouse embryonic fibroblast cells overexpressing ErbB2 (FAK^+/+^-ErbB2 and FAK^−/−^-ErbB2, respectively) (Figure 1A). Herceptin can target human ErbB2 overexpressed in mouse fibroblasts in human cells (Mandler *et al*, 2004, and unpublished data). The invasive capacity of FAK^−/−^-ErbB2 cells was slightly increased compared with FAK-deficient cells, but remained significantly lower than FAK^+/+^-ErbB2 cells (*P*<0.01) (Figure 2C). Herceptin was less effective in FAK^−/−^-ErbB2 cells as compared with FAK^+/+^-ErbB2 cells. Interestingly, re-expression of wild-type FAK restored the inhibitory activity of Herceptin in FAK^−/−^-ErbB2 cells to a level that approached FAK^+/+^-ErbB2 cells (Figure 2C). In a similar manner as the cell invasion assay, the requirement of FAK was also observed for Herceptin-induced inhibition of cell migration on the wound-healing assay (Figure 2C).

To reinforce the connection between FAK and ErbB2 signalling in relation to cell invasion, we showed that the anti-invasive activity following modulation of ErbB2 signalling by Herceptin is similar to the inhibitory effect obtained when FAK expression was downregulated by siRNA in MDA231-ErbB2 cells (Figure 2D). In contrast, inhibition of Akt1 by siRNA to a level that is equal or greater following Herceptin treatment had only a modest but not statistically significant inhibitory effect on cell invasion (Figure 2D). These data support the requirement of FAK for Herceptin-induced anti-invasive activity in ErbB2-positive cells.

### ErbB signalling regulates focal adhesion turnover in an FAK- and Src-dependent manner

**Figure 3:**
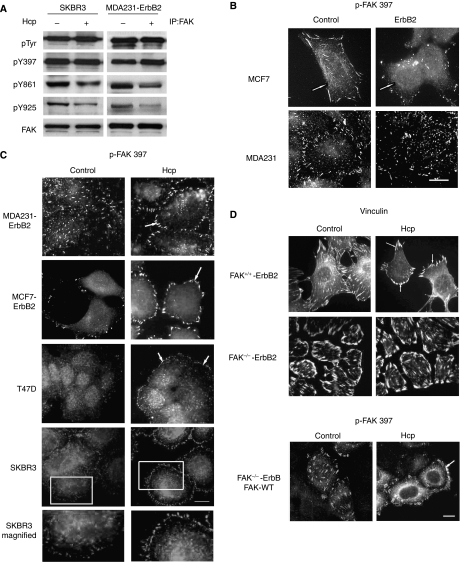
Herceptin induces downregulation of FAK phosphorylation at Y861 and Y925, and reorganisation of focal adhesions at cell membrane. (**A**) SKBR3 and MDA231-ErbB2 cells at 70% confluence were serum-starved for 24 h, treated with IgG1 (−) or 5 *μ*g ml^−1^ Herceptin (Hcp) (+) for 1 h and then stimulated with 10 ng ml^−1^ HRG *β*1 for 10 min. Equal amounts of proteins were immunoprecipitated with anti-FAK. Blots were probed with antibodies against phosphotyrosine, phospho-FAK-Y397, phospho-FAK-Y861, phospho-FAK-Y925, and FAK. (**B**) Cells were fixed and immunostained with phospho-FAK-397, a marker of FAs. Control cells showed larger, elongated FAs at the cells periphery. In contrast, ErbB2 overexpressing cells showed punctuate phospho-FAK397-containing adhesions. White arrows show the position of adhesive contact sites. Scale bar, 30 *μ*m. (**C**) Cells were treated as described in (**A**) and then fixed and immunostained with phospho-FAK-397. Note that control cells exhibit a homogenous subcellular localisation of FAs, whereas in cells treated with Herceptin, FAs are prominently localised at the periphery of cell protrusions (ring-like shape) as shown in the enlargements of the boxed regions. Scale bar, 30 *μ*m. **(D)** FAK^+/+^-ErbB2 and FAK^−/−^-ErbB2 cells were treated under similar conditions as in (**A**). Immunofluorescence labelling of phospho-FAK-Y397 or vinculin showed that under Herceptin treatment, FAK^+/+^-ErbB2 cells exhibited larger FAs within the cell protrusions, whereas the FAK^−/−^-ErbB2 cells remained unchanged with large vinculin-containing adhesions. In contrast, the restoration of wild-type FAK in FAK^−/−^-ErbB2 cells exhibited a robust staining of phospho-FAK-Y397 at the cell protrusions under Herceptin treatment compared with the control, where the phospho-FAK-Y397 is localised at small and numerous focal contacts. Scale bar, 30 *μ*m.

Focal adhesion kinase is an adaptor protein that undergoes multiple phosphorylations and protein–protein interactions. As shown in Figure 3A, exposure of serum-starved MDA231-ErbB2 and SKBR3 cells to 5 *μ*g ml^−1^ Herceptin for 1 h followed by 15 min stimulation with HRG *β*1 had no clear effect on FAK phosphorylation at tyrosine 397; however, reduced phosphorylation was observed at tyrosines 861 and 925.

However, immunofluorescence microscopy showed significant differences in the appearance and distribution of FAs in ErbB2 overexpressing cells, as compared with parental cells. As shown in Figure 3B, control cells formed large and elongated FAs (shown by phospho-FAK Y397, an activation site of FAK that becomes phosphorylated upon FA assembly) that were most prominent at the cell periphery. In contrast, cells that overexpressed ErbB2 formed small and short punctuate phospho-FAK Y397 containing adhesions. These finding suggest that ErbB2 signalling plays a role in the regulation of FA dynamics.

To determine whether ErbB2 signalling is important for FA disassembly, we examined the localisation and appearance of FAs following ErbB2 modulation by Herceptin. As shown in Figure 3C, compared with ErbB2-positive cells treated with IgG1, exposure to 5 *μ*g ml^−1^ Herceptin for 1 h clearly induced changes in the appearance of peripherally localised FAs, such as phospho-FAK Y397, paxillin, and vinculin (data not shown), which increased in number and size and became homogeneously distributed along the plasma membrane. As noted for cell invasion, Herceptin did not affect FAs in cells lacking FAK (Figure 3D), but restoration of FAK in these cells rescued Herceptin activity on FAs resulting in a similar appearance as in FAK^+/+^-ErbB2 cells.

**Figure 4:**
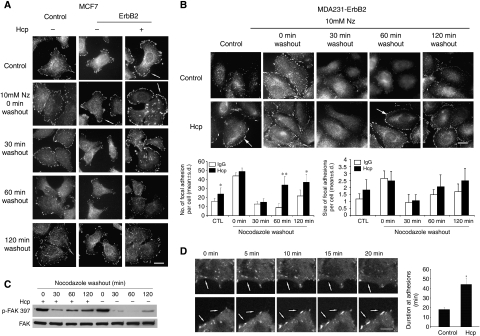
Herceptin regulates the turnover of focal adhesions. (**A**) Serum-starved control MCF7 and ErbB2 cells were incubated with mouse IgG1 (−) or 5 *μ*g ml^−1^ Herceptin (+) for 1 h followed by 10 *μ*M nocodazole (NZ) treatment for 4 h. At the indicated time after nocodazole washout, cells were stained with anti-phospho-FAK-Y397 antibody. Control serum-starved cells exhibit few punctuate FAs, which localise around the cell periphery in the presence of Herceptin. Exposure to nocodazole increased the number of FAs (0 min) followed by a rapid disassembly and then reassembly after nocodazole washout (30 and 60 min). Note that MCF7-ErbB2 cells showed more rapid loss of p-FAK397, as compared with MCF7 control cells, which showed prominent and stable focal adhesions (30 and 60 min). Notably, exposure of MCF7-ErbB2 cells to 5 *μ*g ml^−1^ Herceptin further induced stable FAs (30 and 60 min after nocodazole washout), as compared with IgG1-treated control cells. Scale bar, 30 *μ*m. (**B**) Top panel: serum-starved MDA231-ErbB2 cells taken from duplicate conditions described in (**A**) were immunolabeled with anti-phospho-FAK-Y397 and anti-vinculin antibodies simultaneously. Note that Herceptin induced a more rapid recovery of FAs compared with control cells (60 min). Scale bar, 30 *μ*m. Lower panel: quantification of number and size of focal adhesion by Volocity imaging software. Quantified data were obtained from an average of 20 cells of each condition from three independent experiments. Treatment of Herceptin increased both the number and size of focal adhesions when compared with control cells (^*^P<0.05; ^**^P<0.001). (**C**) Representative western blot of focal adhesion disassembly assay. Serum-starved MDA 231 ErbB2 cells were incubated for 4 h with 10 *μ*M nocodazole (NZ) treatment, and the drug was then washed out and microtubules were allowed to regrowth. Cell lysates were prepared at the indicated times as in (**B**) and separated by SDS–PAGE and western blot was performed. Immunoblotting with the corresponding antibodies showed that the levels of p-FAK-Y397 decreased after nocodazole washout and were roughly correlated with the loss of focal adhesions detected by immunofluorescence. Herceptin-treated MDA231 ErbB2 cells showed more rapid recovery of p-FAK-Y397 compared with control cells. (**D**) Left panel: Herceptin-A induced stabilisation of focal adhesions in real time live cells. BT20 cells stably expressing EYFP-paxillin were plated on fibronectin-coated multi-well chamber coverslides. Video sequences of magnified regions of cells treated with either IgG1 (control) or 5 *μ*g ml^−1^ Herceptin (Hcp) were monitored at 5 min intervals (*t*=0, 5, 10, 15, 20 min). Focal adhesions containing EYFP-paxillin were larger, and more peripheral and stable in cells treated with Herceptin compared with IgG-treated control cells, which have smaller and more dynamic EYFP-paxillin containing adhesions. White arrows show the position of stabilised focal adhesion sites. Scale bar, 10 *μ*m. (see Supplementary data for the related real-time movie). Right panel: quantification of the persistence of EYFP-paxillin in adhesion complexes shows that paxillin persists for longer durations upon incubation of Herceptin. Duration of measurements were made by counting the amount of time lapsed between the first and last frames in which an individual adhesion was observed. Quantifications show the mean±s.d. (^*^P<0.001).

The reorganisation and increased density and size of FAs seen at the cell membrane following inhibition of ErbB2 signalling by Herceptin suggests a change in FA turnover. To address this hypothesis, we used the the nocodazole-based assay (Ezratty *et al*, 2005). Treatment of cells with the microtubule-disrupting agent nocodazole inhibits microtubule targeting and focal complex disassembly. After nocodazole washout, microtubule targeting of focal complexes occurs and promotes focal complex turnover and cell spreading. As shown in Figure 4A, exposure of serum-starved MCF7 cells to nocodazole induced strong FA formation followed by disassembly and then reassembly after nocodazole washout. Interestingly, MCF7-ErbB2 cells showed more rapid loss of phospho-FAK Y397 compared with MCF7 control cells, which had more prominent and stable FAs (30 and 60 min after nocodazole washout). We then examined how ErbB2 modulates the organisation and disassembly of FAs after Herceptin stimulation. We observed that exposure of MCF7-ErbB2 cells to 5 *μ*g ml^−1^ Herceptin induced more stable (30 and 60 min after nocodazole washout) FAs, as compared with IgG1-treated control cells. In contrast, there was no significant change in FAs disassembly following Herceptin treatment in the parental MCF7 ErbB2-negative cells (data not shown).

Under similar conditions, serum-starved MDA231-ErbB2 cells have reduced FAs as shown by phospho-FAK 397 staining (Figure 4B). Exposure of these cells to nocodazole increased FA formation followed by a rapid disassembly (30 min), following nocodazole washout as seen in the control. Focal adhesion reform and appear larger and prominent. Exposure of these cells to 5 *μ*g ml^−1^ Herceptin induced a more stable and rapid recovery (60 min) of FAs, as compared with IgG1-treated control cells (Figure 4B). The number and size of FAs formed under each condition was quantified using Volocity software. Quantified data were obtained from an average of 20 cells of each condition from three independent experiments. Herceptin treatment increased the number of FA by approximately 60% (*P*<0.05) at the control basal level and 120 min after nocodazole washout, and became approximately three-fold at 60 min after nocodazole washout (*P*<0.001). At the same time, Herceptin treatment also slightly increased the size of FAs.

To check biochemically for FA disassembly, we monitored levels of FAK-pY397 by western blot (Figure 4C). The levels of FAK-pY397 decreased after nocodazole washout and were roughly correlated with the loss of FAs detected by immunofluorescence (Figure 4B). The more rapid recovery of FAK-pY397 in the Herceptin-treated MDA231 ErbB2 cells were also observed in the biochemistry (plastic) substrate.

The difference in the kinetics of FA turnover between MCF7-ErbB2 and MDA231-ErbB2 is likely due to the genetic background of these cells. As reported in the Supplementary data Figure 1A, ErbB2 signalling does not affect microtubule organisation. In addition, the differential effect of Herceptin on FA turnover is not due to a possible differential regulation of RhoA, as the expression level of activated RhoA-GTPase in MDA231-ErbB2 was not affected by Herceptin treatment (Supplementary data, Figure 1B; the average densitometry ratios of active RhoA/RhoA were: 1.37±0.21 and 1.43±0.19 for cells collected immediately after nocodazole treatment (0 min) in the absence and presence of Herceptin, and 1.51±0.22 and 1.27±0.19 for cells collected 60 min after nocodazole washout in the absence and presence of Herceptin, respectively). Moreover, similar data on Herceptin-mediated regulation of FA turnover was observed in T47D cells, a breast cancer cell line that express intermediate endogenous ErbB2 (Supplementary data Figure 1C).

The above results on the regulation of FA turnover by ErbB2 signalling were also confirmed using time-lapse video microscopy on BT20 breast cancer cells (these cells express high level of endogenous ErbB2 and are responsive to Herceptin, Yakes *et al*, 2002; Nagata *et al*, 2004) transfected with EYFP-paxillin. As shown in Figure 4D, FAs containing EYFP-paxillin became larger, more peripheral and stable in cells treated with Herceptin compared with control cells treated with IgG1 (a related video is included in Supplementary data). We measured the duration of EYFP-paxillin intensity in forming or disassembling FAs of control and Herceptin-treated cells (Figure 4D right panel) as performed by Franco *et al* (2004). EYFP-paxillin in these cells showed the persistence of paxillin in adhesion complex for extended durations in the treatment of Herceptin. This further established a regulation of FA turnover following modulation of ErbB2 signalling by Herceptin in alternative ErbB2-positive cancer cells.

**Figure 5:**
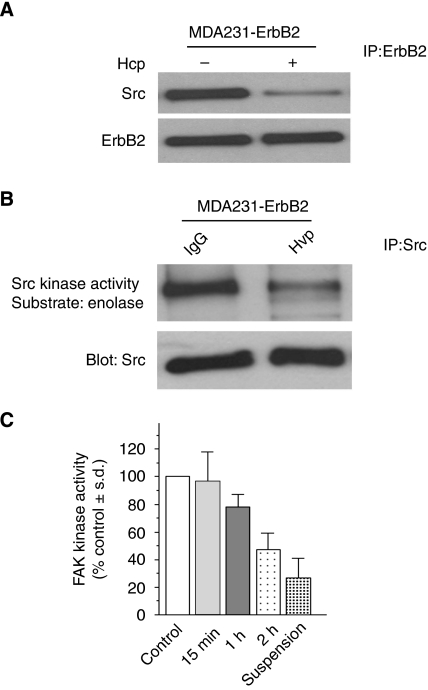
Herceptin inhibits ErbB2–Src interaction, Src kinase activity, and FAK kinase activity. (**A**) Herceptin induces Src dissociation from ErbB2. MDA231-ErbB2 cells were serum-starved for 24 h, stimulated with 10 ng ml^−1^ HRG *β*1 and then treated with IgG1 (−) or 5 *μ*g ml^−1^ Herceptin (Hcp) (+) for 1 h. Anti-ErbB2 immunoprecipitates were blotted with Src antibody to detect ErbB2 bound to Src. The membrane was then stripped and reprobed with anti-ErbB2 antibody. (**B**) Herceptin inhibits Src kinase activity. Cell lysates from cells collected under similar condition as in (**A**) were immunoprecipitated with Src antibody. Immunocomplexes were subjected to Src kinase assays using enolase as a substrate (top panel). The levels of Src used in the kinase assay were monitored by immunoblotting of immunoprecipitates with anti-Src antibody (lower panel). (**C**) Herceptin decreases FAK activity in a time-dependent manner. MDA231-ErbB2 cells were serum-starved for 24 h, stimulated with 10 ng ml^−1^ HRG *β*1 and then kept in suspension or in monolayer treated with IgG1(control) or 5 *μ*g ml^−1^ Herceptin for the indicated times. Cell lysates were used for the FAK assay as described in Materials and Methods. Each bar represents the average of three independent experiments±s.d.

Focal adhesion turnover is regulated through an assembly and disassembly process, where the Src–FAK complex plays an essential role (Webb *et al*, 2004; Yeo *et al*, 2006). ErbB2 interacts with cytoplasmic c-Src (Belsches-Jablonski *et al*, 2001). It is the dual FAK–Src complex that regulates subcellular localisation of FA proteins at cell protrusions (Richardson *et al*, 1997; Schaller *et al*, 1999). On the basis of these observations, we sought to examine if Src plays a role in Herceptin-induced regulation of FAs in ErbB2-positive breast cancer cells. It has been reported that Herceptin treatment induced a rapid dissociation of Src from ErbB2, leading to inhibition of Src kinase activity (Nagata *et al*, 2004). Focusing on MDA231-ErbB2 cells, we observed that treatment with 5 *μ*g ml^−1^ Herceptin reduced ErbB2/Src interaction (Figure 5A) and Src kinase activity (Figure 5B). Interestingly, Herceptin also reduced FAK activity in a time-dependent manner (Figure 5C).

**Figure 6:**
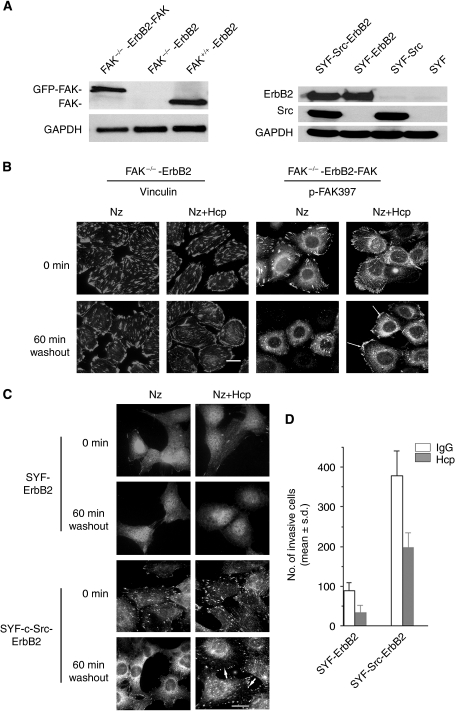
Herceptin-induced regulation of FA turnover is FAK and Src dependent. (**A**) Left panel: immunoblotting of FAK in FAK^−/−^-ErbB2, and their respective FAK-reconstituted cells, and FAK^+/+^-ErbB2 cells. Right panel: SYF cells (Src, Yes, and Fyn triple knockout) and Src-reconstituted SYF cells (SYF-c-Src) were engineered to express ErbB2. Cell lysates from control and ErbB2-expressing cells were used to confirm ErbB2 and Src expression by immunoblotting. As an internal control GAPDH was used . (**B**) Herceptin fails to regulate FA turnover in FAK-deficient cells overexpressing ErbB2 (FAK^−/−^-ErbB2). Cells were treated with mouse IgG1 (−) or 5 *μ*g ml^−1^ Herceptin (Hcp) (+) for 1 h followed by 10 *μ*M nocodazole (NZ) treatment for 4 h (0 min). Cells were then cultured in nocodazole-free medium for the indicated times. Note that FAK^−/−^-ErbB2 are resistant to microtubule-induced disassembly of FA after nocodazole washout, and Herceptin had no effect on the turnover (60 min) of FA immunostained with vinculin. In contrast, immunofluorescence labelling of phosphor-FAK-Y397 showed that the restoration of FAK in FAK^−/−^-ErbB2 cells rescued the effect of Herceptin on FAs assembly (60 min). Scale bar, 30 *μ*m. (**C**) Herceptin failed to regulate FA turnover in SYF-ErbB2 cells. Similar vinculin staining was shown in the absence or presence of Herceptin (Hcp) at 0 and 60 min after nocadazole (NZ) washout. In contrast, Herceptin was able to regulate FA turnover (60 min) in Src-reconstituted SYF-ErbB2 (SYF-Src-ErbB2) cells. Scale bar, 30 *μ*m. (**D**) Herceptin treatment was more potent in inhibiting invasion of SYF-Src-ErbB2 compared with SYF-ErbB2. Cell invasion was determined using the Boyden chamber assay as described in Materials and Methods. Results are expressed as the mean of at least three experiments and three fields per condition±s.d.

To confirm that the regulation of FA turnover by ErbB2 and Herceptin is dependent upon FAK and Src, we examined FA turnover in matched ErbB2-FAK-deficient and ErbB2-Src-deficient cells and their respective reconstituted cells (Figure 6A). Microtubule regrowth failed to induce FA disassembly in FAK^−/−^ cells, and Herceptin failed to regulate FA turnover as shown with vinculin staining. However, microtubule regrowth did induce FA disassembly in FAK^−/−^ cells where FAK is re-expressed. Also Herceptin regained its effect on FA turnover with rapid recovery of FAs compared with control IgG1-treated cells (Figure 6B). Therefore, these results indicate that FAK is required for both microtubule-induced FA disassembly and Herceptin-mediated FA regulation.

In a similar manner, we examined the impact of Src on Herceptin-induced regulation of FA turnover using the SYF cells (triple knockout for Src, Yes, and Fyn) and Src-reconstituted SYF cells engineered to overexpress ErbB2 (Figure 6A). In SYF cells, although FA disassembly occurred normally (Ezratty *et al*, 2005), FA regeneration was defective (Yeo *et al*, 2006). This defect in FA reassembly is consistent with Src being essential to the activation of FAK, which is essential for FA regulation. As shown in Figure 6C, although FA disassembly occurred normally, FA regeneration at 60 min and 2 h (not shown) was defective in SYF-ErbB2 cells, and Herceptin fails to regulate FA assembly and disassembly using phosphor-FAK-Y397 as a marker for FA. However, when Src is re-expressed in SYF-ErbB2 cells, FA disassembly and reassembly occurred at corresponding time points, and Herceptin gained its function as an FA stabiliser in Src-reconstituted cells (Figure 6C). These results correlate with the inhibitory effect of Herceptin on cell invasion (Figure 6D). Moreover, we observed that cell invasion was significantly enhanced in ErbB2-positive and Src-reconstituted cells as compared with Src-deficient cells. Although Herceptin activity was clearly Src and FAK dependent, we noted that Herceptin was still able to slightly reduce the invasion of the non-reconstituted SYF-ErbB2 cells (Figure 6D).

## Discussion

Mounting experimental and clinical evidence supports that primary breast cancer dissemination to distant organs is an early genetic event that can occur during cancer development (Weigelt *et al*, 2005). The early cancer cell migration involves a network of FA and cystoskeletal proteins regulated by growth factor receptors, integrins, and components of the ECM. Focal adhesions are specialised structures that mediate the formation and extension of finger-like protrusions required for cell attachment, cell contraction, cell migration, and invasion of neighbouring structures. One of the key proteins involved in this dynamic process is FAK, which in conjunction with partners of the FAs network couples growth factor receptors and integrins to FAs and regulates early cell migration. Focal adhesion kinase is found to play a critical role in oncogenic transformation and invasion induced by members of the ErbB family (Sieg *et al*, 2000; Benlimame *et al*, 2005) as well as chemical carcinogens (McLean *et al*, 2004).

In this study, we document that in ErbB2-positive invasive breast cancer cells FA disassembly is rapid compared with ErbB2 negative cells where FAs are more stable and prominent at the cell periphery. This supports an important function of ErbB2 signalling in the regulation of FA turnover in invasive breast cancer cells. Herceptin binds to the juxtamembrane region of the ErbB2 ectodomain (Cho *et al*, 2003), a region involved in receptor dimerisation and transmembrane signalling. Noticeable, the antiproliferative activity of Herceptin on ErbB2-positive breast cancer cells is minimal compared with its drastic anti-invasive activity. Several debated mechanisms have been reported for the therapeutic activity of Herceptin. For instance, Herceptin has been reported to induce downregulation of ErbB2 receptor activity by promoting its recycling, internalisation, and/or lysosomal degradation (Klapper *et al*, 2000; Friedman *et al*, 2005); inhibition of ErbB signalling, in particular the PI-3K/Akt pathway (Ignatoski *et al*, 2000; Yakes *et al*, 2002) and activation of PTEN (Nagata *et al*, 2004), leading to inhibition of cell proliferation and enhanced cell susceptibility to apoptosis; decreased cleavage of ErbB2 receptor ectodomain, which prevents the formation of the active truncated form of the receptor (Molina *et al*, 2001); antibody-dependent cell-mediated cytotoxicity by engaging Fc*γ* receptors on immune effector cells through the IgG1 Fc region of Herceptin (Clynes *et al*, 2000); increased chemotherapy-induced DNA damage (You *et al*, 1998; Pietras *et al*, 1999), and indirect antiangiogenic effect by inhibition of angiogenic factor secretion, for example, VEGF, by cancer cells (Yen *et al*, 2000; Izumi *et al*, 2002; Klos *et al*, 2003). The implications of these mechanisms for Herceptin antimetastatic activity *in vivo* remains debated. For instance, a study by Yoeli-Lerner *et al* (2005) showed that activation of Akt, but not its downregulation, inhibits breast cancer invasion through inhibition of the transcription factor NFAT. This study suggests that inhibition of Akt signalling by Herceptin cannot explain Herceptin antimetastatic activity. Indeed, downregulation of Akt by siRNA to a level that is similar to that obtained following Herceptin treatment, only modestly reduced the anti-invasive activity when compared with Herceptin treatment or downregulation of FAK by siRNA. This is in agreement with the study by Yoeli-Lerner *et al* (2005).

The inhibition of FA disassembly by Herceptin, resulted in more stable FA sites at the cell membrane protrusions, in contrast to IgG-treated cells where FA turnover is rapid. This pattern of reduced FA disassembly by Herceptin resembled the appearance of FAs in several cell motility-deficient cells, including FAK^−/−^ cells (Ren *et al*, 2000; Sieg *et al*, 2000; Webb *et al*, 2004) and Src^−/−^ cells (Ilic *et al*, 1995; Cary *et al*, 2002; Yeo *et al*, 2006). Indeed, we showed that Herceptin-induced inhibition of FA disassembly and cell invasion were lost in FAK- and Src-deficient cells overexpressing ErbB2 but are rescued upon re-expression of FAK or Src. Cell invasion signalling occurs through distinct pathways, and in particular FA signalling, which is critical for the formation and turnover of new adhesions at the leading edge of motile cancer cells (Rottner *et al*, 1999; Smilenov *et al*, 1999). The Src–FAK complex has been implicated as a key regulator for FA turnover, namely the rate of adhesion formation, disassembly, and/or maturation (Schaller *et al*, 1994; Fincham and Frame, 1998; Sieg *et al*, 2000; Volberg *et al*, 2001). In particular, Yeo *et al* (2006) and Webb *et al* (2004) have reported a key function for the Src–FAK complex in FA disassembly. Activation of the Src–FAK signalling is generally reported to promote F-actin stress fiber assembly and suppresses RhoA activity (Ren *et al*, 2000); these events can contribute to FA disassembly and enhanced cell migration (Ridley and Hall, 1992; Rottner *et al*, 1999). Our study excludes the possibility that Herceptin-induced changes in FA turnover are mediated by modulation of the Rho GTPase pathway, as no differences were noted in activated RhoA in control *vs* Herceptin-treated cells, in agreement with a study by Webb *et al* (2004) where FA turnover was shown to be independent from RhoA activity.

The FAK–Src signalling has been shown to interrupt the maturation of FAs by promoting their disassembly (Webb *et al*, 2004). This in turn allows continuous adhesion turnover as the cells protrude and migrate. When the Src–FAK signalling is inhibited, adhesion disassembly in protrusive regions is impaired in the presence of Herceptin, similar to Src- and FAK-deficient cells. Our study showes that the exposure of ErbB2-positive cells to Herceptin inhibits Src binding to ErbB2 and Src kinase activity. Under similar condition, FAK activity is inhibited by Herceptin. This novel FAK inhibitory mechanism for Herceptin further establishes a connection between ErbB, Src, and FAK signalling. Therefore, we reasoned that Herceptin, by inhibiting Src and FAK activities, lead to inhibition of FA turnover, which manifest by increased FA stability and reduced cell invasion (a schematic model where Herceptin-mediated regulation of FAs is contrasted with earlier reported mechanisms for Herceptin as shown in Supplementary Figure 2).

However, there is a multitude of additional Src- and FAK-regulated pathways that may account for the regulation of FA turnover by ErbB signalling, which cannot be ruled out at present. For instance, although Src SH2 interaction with FAK is necessary for appropriate recruitment of Src and FAK to FA in migratory cells, the FAK–Src complex can also regulate the phosphorylation of the adaptor proteins paxillin and p130Cas (Bellis *et al*, 1995; Cary *et al*, 1998); the latter can bind independently to both FAK and Src. Phosphorylation of both paxillin and p130Cas creates binding sites for the SH2 domain of the Crk adaptor protein (Iwahara *et al*, 2004), which can impact on the maturation of FA complexes to stable FAs or turn them rapidly (Webb *et al*, 2004; Zaidel-Bar *et al*, 2007). Indeed, cells deficient in p130Cas or paxillin, similar to FAK- and Src-deficient cells, have impaired adhesion disassembly and reduced motility (Webb *et al*, 2004). In contrast to FAK-deficient cells, we noted that SYF-ErbB2 cells acquired a slight and reproducible increase in invasiveness, although to a much lower extent than the Src-reconstituted SYF-ErbB2 cells, and Herceptin reduced invasion of these Src-deficient cells (Figure 6D). This would suggest that ErbB signalling and Herceptin may also regulate cell invasiveness through an Src-independent mechanism(s). These alternative mechanisms for the regulation of FA turnover highlight the clinical potential of inhibitors of FA turnover for the management of invasive cancers. Several investigational drugs, including small molecule kinase inhibitors against FA and its homologue Pyk2 (Roberts *et al*, 2008), are at the stage of early clinical trials and may prove more efficient modulators of FA turnover and hence can prevent the progression of invasive cancers to metastases. This is particularly important as many of the FA proteins, such as ErbB receptors, are overexpressed in invasive cancers (Behmoaram *et al*, 2008).
