# Supplementary figures and images for: Regulation of focal adhesion turnover by ErbB signalling in invasive breast cancer cells
Source: Br J Cancer. 2009 Feb 3;100(4):633–43. doi: 10.1038/sj.bjc.6604901 (PMC2653743; doi:10.1038/sj.bjc.6604901)

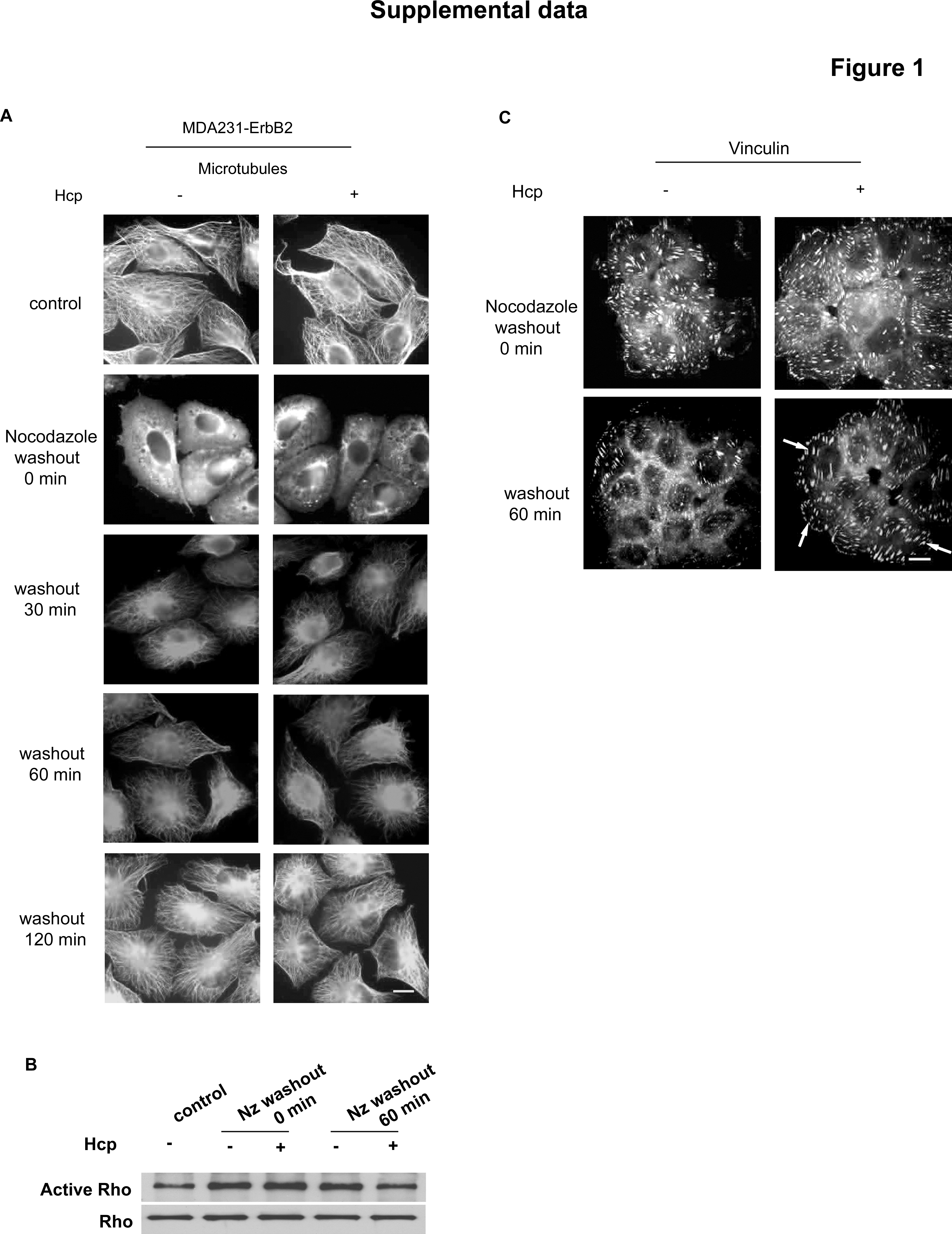

Supplement: Supplementary Figure 1 [file 6604901x1.tif]

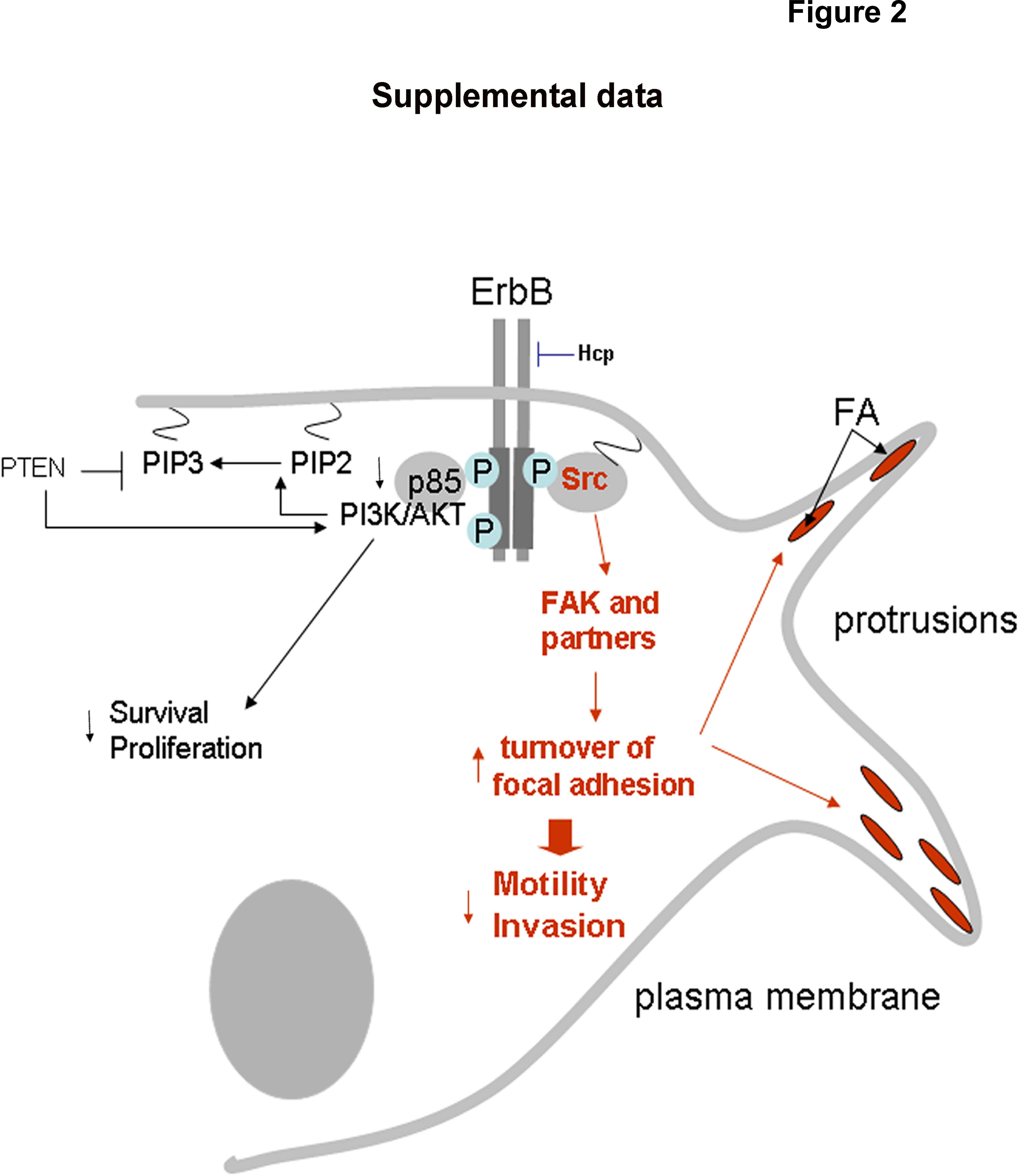

Supplement: Supplementary Figure 2 [file 6604901x2.tif]
