# Supplementary material for: Regulation of focal adhesion turnover by ErbB signalling in invasive breast cancer cells
Source: Br J Cancer. 2009 Feb 3;100(4):633–43. doi: 10.1038/sj.bjc.6604901 (PMC2653743; doi:10.1038/sj.bjc.6604901)
Supplement: Supplementary Data Legends [file 6604901x4.doc]

**Legends for Supplemental data**

**Figure 1** Herceptin regulates focal adhesion turnover

**(A)** Nocodazole induces transient microtubule depolymerization in ErbB-2-positive cells. Serum-starved MDA231-ErbB-2 cells were incubated with mouse IgG1 (-) or 5μg/ml Herceptin (+) for 1 h followed by 10μM nocodazole (NZ) treatment for 4 h. At the indicated time after nocodazole washout, cells were stained with anti-α-tubulin antibody. Note that microtubules started to re-polymerize 30 min after nocodazole washout. Scale bar, 30μm.

**(B)**  RhoA activation is not affected by Herceptin in control and nocodazole-treated cells. MDA231-ErbB2 proteins were taken from duplicate conditions (0 min and 60 min after nocodazole washout) as described in **A** and were used to determine Rho-GTPase activity. Control corresponds to serum starved and non-stimulated cells.

**(C)** Herceptin induces similar changes in the turnover of FA in other ErbB-2 positive cell T47D. Nocodazole treatment induced a significant increase in vinculin-staining within FAs (0 min), followed by a decrease after nocodazole washout. In contrast, Herceptin treatment induced a rapid recovery of FA (60 min). Scale bar, 30μm.

**Figure 2** A schematic model where known mechanisms of Herceptin are contrasted with FA regulation

Activated ErbB receptors couples to several signaling pathways, including the phosphatidylinositol 3-kinase (PI3-K)/Akt pathway and Src. Phosphorylation of tyrosine residues in the intracellular domain of ErbB recruits and activates PI-3K. Activated PI-3K induces the formation of 3-phosphorylated phosphoinositide products (PIP3), which directly or indirectly (through the phosphoinoside-dependent protein kinases (PDK) ) leads to the activation of downstream targets of the PI-3K-Akt pathway; this mechanism can also be contributed through the activation of PTEN. Herceptin (Hcp) induces a rapid downregulation of PI3K/Akt signaling and can relieve PTEN from its inactive state by Src phosphorylation; PTEN can now translocate to the plasma membrane and dephosphorylates PIP3, thereby decreasing the activity of PI-3K/Akt downstream targets. These mechanisms have been implicated primarily for Herceptin-induced inhibition of cell proliferation and induction of apoptosis.

In parallel, activated Src binds to phosphor-Y397 of FAK. This in turn induces the phosphorylation of additional FAK tyrosine kinase sites in the catalytic and C-terminal domains and promotes optimal FAK kinase activity. The Src-FAK complex is critical for the regulation of signaling involved in FA turnover at cell protrusions. Herceptin inhibits ErbB2-interaction with Src and Src kinase activity, as well as Src-induced activation of FAK, which in turn lead to inhibition of FA turnover. This can lead to increased stability of FAs, which in turn can lead to inhibition of cell migration and invasion.

**Video**

BT20 cells expressing EYFP-paxillin were plated on fibronectin coated chamber coverslide. Video sequences of magnified regions of cell treated with 5µg/ml Herceptin was monitored at 1-minute intervals for over 30 minutes.
